# Utilizing profile hidden Markov model databases for discovering viruses from metagenomic data: a comprehensive review

**DOI:** 10.1093/bib/bbae292

**Published:** 2024-06-20

**Authors:** Runzhou Yu, Ziyi Huang, Theo Y C Lam, Yanni Sun

**Affiliations:** Department of Electrical Engineering, City University of Hong Kong, Tat Chee Avenue, Kowloon, Hong Kong, China; Department of Electrical Engineering, City University of Hong Kong, Tat Chee Avenue, Kowloon, Hong Kong, China; Department of Electrical Engineering, City University of Hong Kong, Tat Chee Avenue, Kowloon, Hong Kong, China; Department of Electrical Engineering, City University of Hong Kong, Tat Chee Avenue, Kowloon, Hong Kong, China

**Keywords:** profile hidden Markov models, virus detection, metagenomic data

## Abstract

Profile hidden Markov models (pHMMs) are able to achieve high sensitivity in remote homology search, making them popular choices for detecting novel or highly diverged viruses in metagenomic data. However, many existing pHMM databases have different design focuses, making it difficult for users to decide the proper one to use. In this review, we provide a thorough evaluation and comparison for multiple commonly used profile HMM databases for viral sequence discovery in metagenomic data. We characterized the databases by comparing their sizes, their taxonomic coverage, and the properties of their models using quantitative metrics. Subsequently, we assessed their performance in virus identification across multiple application scenarios, utilizing both simulated and real metagenomic data. We aim to offer researchers a thorough and critical assessment of the strengths and limitations of different databases. Furthermore, based on the experimental results obtained from the simulated and real metagenomic data, we provided practical suggestions for users to optimize their use of pHMM databases, thus enhancing the quality and reliability of their findings in the field of viral metagenomics.

## Introduction

The application of metagenomic sequencing, particularly viral metagenomics, has greatly enhanced our comprehension of viromes across diverse ecosystems in recent years [1–3]. Metagenomic sequencing’s ability to sequence all genetic material enables us to comprehensively profile the viral constituents within each sample. There is a suite of tools available for identifying or validating viruses from metagenomic data [4–9]. A very important step in these tools’ operation is the alignment of sequences against a database of profile hidden Markov models (pHMMs) to detect and characterize viruses. Some tools rely on the pHMM matches to identify viral contigs and conduct viral taxonomic classification [7]. Some other tools use the pHMM matches as important features in a machine learning model for virus contig classification [5]. The underlying viral protein domains or families that can be used as markers for viruses or a specific viral group help the virus discovery and taxonomic analysis.

Profile HMM is one of the most prevalent models for biological sequence analysis [10, 11]. Built from the multiple sequence alignment (MSA) of a group of homologous sequences, pHMM is able to encode the position-specific mutations/insertions/deletions. Then, available decoding algorithms such as Viterbi can be applied to compute the probability that a query sequence is generated by a pHMM, thus allowing users to determine whether the query sequence is homologous to the underlying sequence family or domain. pHMM-based alignment exhibits higher sensitivity for remote homology search than pairwise sequence alignment and is widely used in biological sequence analysis. pHMMs play important roles in viral sequence analysis, such as detecting and classifying viral sequences, identifying conserved regions, and annotating functional elements within viral genomes [12–15]. pHMMs can also be used for identifying sequence similarities across different viruses down to the strain level [16]. This is particularly useful for studying viral evolution, identifying genetic variations, and tracking the spread of viral outbreaks.

A diverse range of pHMM databases are available to support virus research. These databases serve different purposes, with some offering comprehensive coverage across various taxonomic groups, such as Pfam [17], KOfam [18], and SuperFamily [19]. Other databases are specifically dedicated to viruses, providing valuable resources for viral research, such as vfam [13], VOGDB [20], and pVOGs [15]. Additionally, certain databases such as EggNOG [21, 22] offer entry points for viral proteins, further facilitating analysis and exploration within the viral protein domain. These databases vary significantly in terms of the quantity of models they contain, the conservation level of the underlying sequences, and their effectiveness in virus detection. Despite these differences, a thorough review comparing these pHMM databases for virus detection in metagenomic data is currently absent.

In this study, we present a comprehensive review of the application of pHMMs in detecting viral sequences from metagenomic data. We scrutinize the design logic and basic statistics of various databases, devise a uniform metric to assess model conservation, and offer practical guidance on selecting suitable databases for specific applications. Furthermore, we extensively evaluate their performance in identifying viral sequences from metagenomic data, using both simulated and actual sequencing data.

## Review of the current pHMM databases

The construction of a pHMM database generally follows four key steps.

- Collection of training or seed sequences. The initial step involves collecting the “training/seed” sequences that will be utilized to construct the model. The difference in the data source can influence the resulting databases.
- Sequence clustering and alignment. Sequences are then grouped into families so that each family contains sequences with high sequence similarity, which implies similar structures or functions. This grouping can be achieved using methods such as clustering or iterative alignment. Subsequently, the sequences within a family are aligned using MSA algorithms, such as MUSCLE [23].
- Construction of HMM. The third step entails constructing a pHMM from the MSA. Tools such as the ’‘hmmbuild” module in HMMER [10] are often employed for this purpose.
- Family specific curation. This step adjusts the model’s scoring system to differentiate true matches from background noise or random matches. For example, Pfam designs GA cutoff. CheckV [6] chooses virus-specific pHMMs by examining the number of alignments against viral proteins and non-viral proteins for each pHMM. VIRify [4] assigns family-specific bit score cutoffs for target taxonomic groups.

The choice of the data source, the method of identifying a group of homologous sequences, and the parameters in the alignment step can all lead to differences in the database, such as the number of models and the taxonomic coverage of the sequences in one model.

### pHMM databases for virus detection

We are mainly interested in reviewing databases that can be used to identify viruses. Table 1 contains major pHMM databases that cover viruses. We will review their construction process, compare their scale, and evaluate their model-specific taxonomic distribution patterns using an entropy-based metric. Then, we will examine their empirical performance on virus identification in several application scenarios.

**Table 1:** Overview of commonly used pHMM databases that can be used for virus classification; the “Virus Type” column specifies the categories of viruses each database is designed for: “Pro” for prokaryotic viruses, “Eu” for eukaryotic viruses, and “All” for all taxonomic groups including and beyond viruses; the “Training Seq Access” column indicates the accessibility of the training sequences used to construct the pHMMs in each database; without causing any ambiguity, EggNOG-4.5-Virus and EggNOG-5.0-Virus are shortened as EggNOG-4.5 and EggNOG5.0 hereafter

**Database**	Publish Year	Reviewed Version	**#models**	**Model Availability**	Virus Type	Training Seq Access
**EggNOG-4.5-Virus**	2015	–	998	http://eggnog45.embl.de/#app/viruses	Pro & Eu	N
**EggNOG-5.0-Virus**	2018	–	8318	http://eggnog5.embl.de/download/eggnog_5.0/per_tax_level/10239	Pro & Eu	N
**vFam**	2014	–	5585	https://derisilab.ucsf.edu/software/vFam/	Eu	Y
**pVOGs**	2017	–	9518	https://ftp.ncbi.nlm.nih.gov/pub/kristensen/pVOGs	Pro	Y
**Pfam**	1997	Pfam 35.0	19 632	https://www.ebi.ac.uk/interpro/download/Pfam/	all	Y
**KOfam**	2019	2023	25 267	https://www.genome.jp/tools/kofamkoala/	all	N
**VOGDB**	2022	vog216	35 185	https://vogdb.org/	Pro & Eu	Y
**SuperFamily**	2001	–	7924	https://supfam.org	all	N

**EggNOG** [21] is a comprehensive resource for studying orthology and function across diverse organisms. The acronym “EggNOG” stands for “Evolutionary genealogy of genes: Non-supervised Orthologous Groups.” It provides a hierarchical classification of genes into orthologous groups.

The main purpose of the EggNOG database is to assign functional annotations to genes based on their orthology relationships. By analyzing orthology, EggNOG can infer the potential functions of uncharacterized genes based on the functions of their orthologs. This is particularly useful for newly sequenced or poorly studied organisms. In EggNOG, viral proteins are obtained from all UniProt reference proteomes and filtered via quality control. Some proteomes are also manually reviewed before analysis.

We focused on EggNOG 4.5 [24], the first version incorporating viral orthologous groups, expanding coverage beyond cellular life. All viral models from this version were used. We also included EggNOG 5.0 [22] due to its substantial updates, expanding from 352 to 2502 proteomes. However, EggNOG 6.0 [25] lacks organism-specific downloads so was excluded.

The comprehensive EggNOG database encompasses bacteria, archaea, eukaryotes, and viruses. EggNOG 4.5 covers 2031 prokaryotic/eukaryotic organisms and over 352 viral proteomes. EggNOG 5.0 expanded to include 5090 organisms and 2502 viruses.

**vFam** [13] is a collection of 5585 profile HMMs constructed from 29 655 viral proteins of eukaryotic viruses in the RefSeq database, last updated in 2014. vFam stands for “Viral Families,” and this database focuses specifically on capturing and cataloging the diversity of viral proteins and their functional domains. The primary purpose of the vFam pHMM database is to provide researchers with a comprehensive collection of HMMs that represent different viral protein families.

**pVOGs** [15] is the Prokaryotic Virus Orthologous Groups database, focusing on bacteriophages and archaeal viruses. It represents a comprehensive collection of orthologous gene families extracted from complete viral genomes. The construction of pVOGs adopts the Clusters of Orthologous Groups framework, which is widely used for prokaryotic orthology identification.

The latest pVOGs release contains 9518 orthologous groups shared among nearly 3000 complete genomes of prokaryotic viruses. This expanded pVOGs collection has broad applicability for taxonomy, evolutionary genetics, genomic epidemiology, metagenomics, systems biology, ecology, and more.

**Pfam** [26] is one of the most comprehensive and earliest profile HMM databases for encoding a large number of protein families or domains. Each entry within the Pfam database represents a protein domain/family and is identified by a unique identifier (Pfam ID). It includes a description of the family’s function and properties. These families undergo meticulous curation and classification, considering sequence similarity, protein structure, and functional information.

Pfam is regularly updated to incorporate new protein sequences, enhancing the accuracy and coverage of its annotations. It offers a user-friendly web interface enabling users to search for specific protein families, access detailed protein family information, and perform sequence searches within the database.

**KOfam** [18] database, short for Kyoto Encyclopedia of Genes and Genomes (KEGG Orthology-Based Annotation System), provides a comprehensive collection of pHMMs that represent specific protein domains or functional motifs. These pHMMs are constructed based on the KEGG Orthology (KO) database [27], which organizes genes and proteins into functional categories and pathways. An advantage of the KOfam database is that it is updated regularly to incorporate the latest information from the comprehensive KEGG Orthology database and reflects the advancements in functional annotation. It also offers a user-friendly interface and there are various search tools such as KofamKOALA and KofamScan [18], allowing researchers to query and analyze protein sequences using the KOfam HMMs. Additionally, the database provides statistical scores and confidence values to assess the reliability of the annotations.

**VOGDB** [20], short for Virus Orthologous Groups Database, is a continuously updated resource based on all RefSeq virus genomes, and thus, it can represent all viral lineages that have available complete genomes. At the time of writing, the latest release of VOGDB on 27 August 2023, encompassing 12 590 genomes and 603 216 proteins, contains 38 161 virus orthologous groups (VOGs). VOGDB provides virus-specific groups. This allows us to identify viral sequences in metagenomes or pro-viruses in cellular (meta) genomes [28].

**SuperFamily** [29] (supfam) is a comprehensive database and resource for exploring protein superfamilies, which consist of related protein domains with similar structures and functions. The database includes information on domain architectures and functional annotation. It is valuable for studying protein structure–function relationships, predicting protein functions, and exploring the evolutionary history of protein domains. SuperFamily 1.0 was released in 2001 and contained over 7000 models. SuperFamily 2.0 [19] was released in 2019, containing a total of 27 623 pHMMs. Because Supfam builds pHMMs based on proteins of known structures, it is not an ideal choice for finding disordered proteins or families that are unrelated to proteins of known structures. Additionally, applying SuperFamily for viral sequence detection involves non-trivial additional work. Therefore, we only look at the metadata of this database and did not include it in subsequent experiments.

In addition to the reviewed databases, other pHMM databases exist but were excluded for various reasons. For instance, Dfam [30] stores models representing repetitive DNA elements present in eukaryotic genomes, rendering it unsuitable for viral sequence detection. PHROG [31] constructs models for prokaryotic virus proteins using the HHsuite format [32] rather than the HMMER format used in our analysis. Therefore, PHROG was omitted from the comparative evaluation.

### Basic statistics of different databases

**Figure 1:**
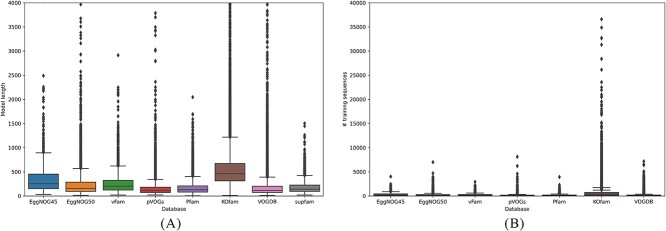
Basic information of all pHMMs in eight pHMM databases: (A) the distribution of model length for eight databases; median values of model lengths in each database are 255 (EggNOG45), 160 (EggNOG50), 207 (vFam), 109 (pVOGs), 129 (Pfam), 461 (KOfam), 116(VOGDB), and 143 (supfam), and outliers beyond 4000 are not displayed; (B) the distribution of the number of training sequences for pHMMs in seven databases; the database SuperFamily is excluded in this figure for not providing this information, and median values of training sequences of pHMMs in each database are 3 (EggNOG45), 2 (EggNOG50), 3 (vFam), 6 (pVOGs), 23 (Pfam), 347 (KOfam), and 3(VOGDB); outliers beyond 40 000 are not displayed.

We summarize the basic facts and statistics of the above databases in Table 1. In general, comprehensive pHMM databases are larger than virus-specific databases. The only exception is VOGDB, which has the largest number of small models in terms of the seed proteins’ length and the number of seed sequences. Figure 1A illustrates the distribution of the model length, which is the number of match states in a model. The match state number is largely proportional to the dominant length of the training sequences underlying a model. Thus, Fig. 1A reflects the length of the underlying protein family/domain in each database. The median model size in most databases is below 300 amino acids. Notably, KOfam stands out as it contains a significant number of models exceeding 500 amino acids in length. On the other hand, the largest database VOGDB hosts a considerable number of small models with model size shorter than 200 amino acids.

Figure 1B illustrates the distribution of the number of training sequences of each single pHMMs from these databases. In Supplementary Fig. S1, we provide more detailed information about the models in each database. We plot the distribution of model lengths (M_len), the distribution of the number of sequences for training (nseq), and the scatter plots between these two factors.

### Taxonomic diversity analysis of pHMMs

Different pHMM databases vary in the degree of conservation in their models, which can influence their potential applications and the efficacy of virus discovery and characterization. Specifically, models featuring highly conserved viral proteins can be utilized for both virus discovery and taxonomic classification because the taxonomic label in a model is consistent. However, these highly conserved models often result from fine-grained clustering strategy. The similarities between related models can lead to ambiguous model matches. Therefore, characterizing the model conservation at different taxonomic ranks can provide important insights into the choice of pHMM database for virus research. Inspired by alpha diversity [33] computation, we have developed a metric to quantify the “taxonomic purity” of a model based on Shannon entropy for each taxonomic rank. For example, a pHMM trained solely with viral sequences would have an entropy of zero at the superkingdom level. Conversely, a pHMM with large entropy at a higher taxonomic rank suggests a greater diversity within the training sequences.

**Figure 2:**
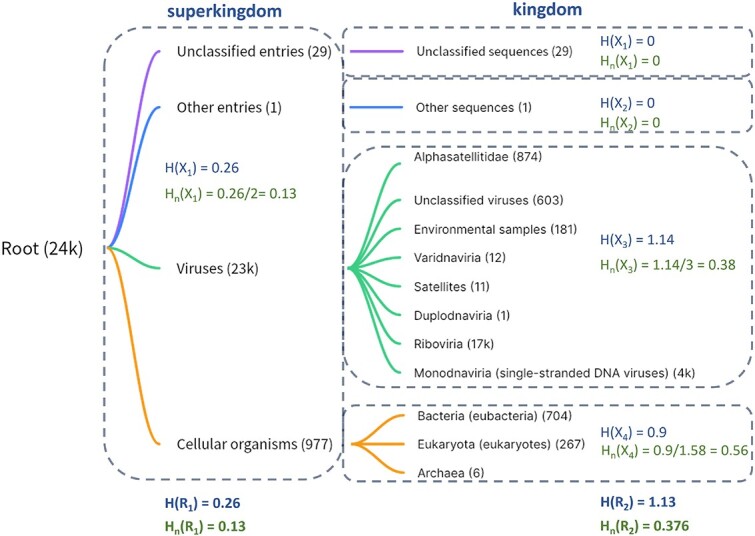
Example of entropy calculation for the taxonomic tree using the training sequences of the Pfam model PF00910; $H(X)$ denotes the original Shannon entropy value; $H_{n}(X)$ denotes the normalized entropy value.

To evaluate the taxonomic purity of a profile HMM, we begin by constructing a taxonomic tree using all the training sequences associated with the model. Each node in the tree is assigned a weight that represents the total number of training sequences within its subtree. Subsequently, we calculate the Shannon entropy for each node. As an example, Supplementary Fig. S2 depicts the taxonomy tree formed by the training sequences of RNA helicase model (PF00910) from Pfam. Due to the limited space, we only expand the dominant branches of this tree, showcasing taxonomic ranks from the superkingdom level to the species level (the last layer is not expanded). In Fig. 2, we show all the nodes in the two highest taxonomic ranks and illustrate the entropy calculation.

**Figure 3:**
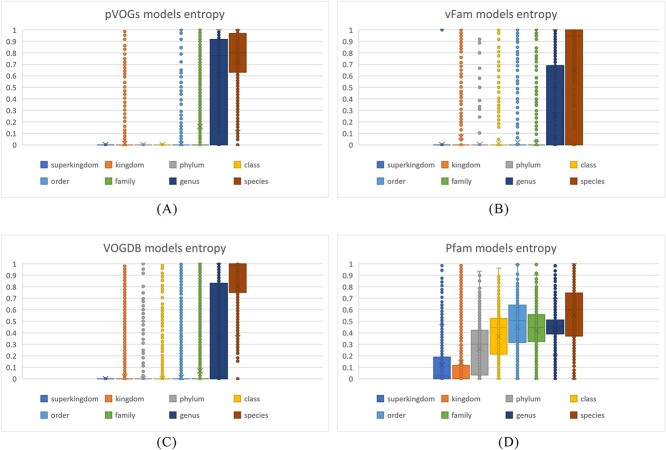
Boxplot of normalized Shannon entropy distribution of four pHMM databases with accessible training sequences and associated taxonomic labels; median entropy of pVOGs: 0.78 (genus), 0.8 (species), and median entropy of vFam: 0 (genus), 0.95 (species); median entropy of VOGDB: 0 (genus), 0.98 (species); median entropy of Pfam: 0.44 (genus), 0.63 (species).

At each rank of a taxonomic tree, we first calculate the entropy of each group $X$, which is a collection of sibling nodes sharing the same parent node. For example, the group $X_{3}$ in Fig. 2 represents the taxonomic distribution at the rank kingdom under the “viruses” node. The entropy associated with a group is computed as follows: 


(1) \begin{align*}& H(X) = -\sum_{i=1}^{k}{p(x_{i})log_{2}p(x_{i})},\end{align*}

where $k$ is the number of sibling nodes in group $X$.

For all groups at the same taxonomic rank, we use their weighted sum as the entropy of this pHMM at this specific rank 


(2) \begin{align*}& H(R) = \sum_{all\ groups\ at\ rank\ R}{P_{X}H(X)},\end{align*}

where $P_{X}$ is the ratio of the number of training sequences in group X to the total number of training sequences.

H(R) provides useful information for describing the conservation level at a specific taxonomic rank R. However, as different groups may have various numbers of sibling nodes, H(R) cannot be directly compared across different node groups or pHMM models. For example, in Fig. 2, the training sequences under “Viruses” node predominantly belong to the “*Riboviria*” clade, while training sequences under “Cellular organisms” are more evenly distributed across different kingdoms. Therefore, the purity of the group under “Viruses” node ($X_{3}$) is expected higher than that of the group under “Cellular organisms” node ($X_{4}$). However, the weighted entropy values for $X_{3}$ and $X_{4}$ are 1.14 and 0.9, respectively, which do not reflect the observed taxonomic distribution patterns.

To mitigate this issue, we apply a normalization step by dividing H(R) by the maximum possible entropy value. The entropy reaches the maximum for a group X with k children when $P_{X_{i}}$ (i=1 to k) is uniform. For example, for $k = 2$, $max(H_{2}) = 1$; for $k = 3$, $max(H_{3}) = 1.58$; for $k = 4$, $max(H_{4}) = 2$; for $k = 8$, $max(H_{8}) = 3$. Thus, the normalized entropy for a node and a taxonomic rank is defined as


(3) \begin{align*}& \begin{aligned} H_{n}(X) &= -\sum_{i=1}^{n}{p(x_{i})log_{2}p(x_{i})}/max(H_{k}) \\ H_{n}(R) &= \sum_{all\ nodes\ at\ rank\ R}{P_{X}H_{n}(X)} \end{aligned}.\end{align*}

The normalized entropy values for $X_{3}$ and $X_{4}$ at kingdom rank are 0.38 and 0.56, respectively, in the example. The adjusted entropy values offer a more accurate reflection of the purity of the taxonomic labels under each node and allow a fair comparison across different models.

Of the reviewed databases, four of them, pVOGs, vFam, Pfam, and VOGDB provide access to their training sequences with associated taxonomic information (or accession numbers that can be linked to taxonomic information). Leveraging these data, we conducted an analysis of entropy for these databases. Supplementary Figure S3 presents the distribution of raw Shannon entropy values for all models within each database. Figure 3 illustrates the distribution of normalized Shannon entropy values for all models within these four databases. These figures offer insights into the entropy characteristics of the models in these databases, shedding light on their diversity and potential applications. It is important to note that the entropy of a model is a composite measure that encompasses entropy values at various taxonomic ranks. A larger entropy value at higher taxonomic ranks indicates that the taxonomic composition of the training sequences is less homogeneous or pure.

Three of the databases (pVOGs, vFam, and VOGDB) specifically focus on viral proteins. These databases exhibit near-zero entropy values at the superkingdom level, indicating that nearly all the training sequences consist of viral proteins. However, the entropy value distribution at other taxonomic ranks varies among these three databases. Notably, all models from pVOGs exhibit zero entropy at the phylum and class ranks, suggesting that the training sequences of pHMMs in pVOGs are from the same class and only diverge beyond the order level. Vfam and VOGDB have 0 median entropy values at the genus level, indicating that their models tend to be trained by sequences from one viral species. In contrast, as a comprehensive database, Pfam demonstrates larger entropy values at higher taxonomic ranks from superkingdom level to genus level compared with the other three databases. The entropy at the species rank is calculated from the distribution of training samples at subspecies level or strain level (any rank below species). Pfam’s models do not contain many different subspecies or clades below species level, leading to smaller composite entropy at the species rank. It is important to note that when assessing the overall entropy or purity of an individual model, the emphasis on different taxonomic ranks should vary. Generally, higher entropy at higher taxonomic ranks suggests a lower level of purity in a pHMM. Supplementary Figure S4 provides a visual representation of the entropy values for the top 10 largest models, based on the number of training sequences, obtained from two databases: vFam (a viral database) and Pfam (a comprehensive database). The entropy values are depicted across all taxonomic ranks. Notably, models from vFam exhibit larger entropy values at the two lowest taxonomic ranks (genus and species). On the other hand, models from Pfam showcase larger entropy values at higher taxonomic ranks.

## Utilizing pHMMs for virus detection

**Figure 4:**
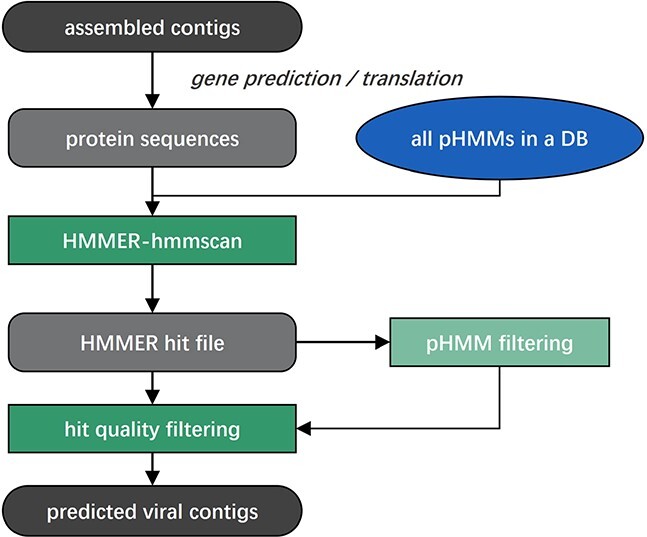
Pipeline for applying pHMM databases to detect viral sequences from metagenomic data; the step pHMM filtering applies E-values or model-specific score cutoffs to distinguish viral contigs from non-viral contigs.

After analyzing the properties and conservation levels of pHMM databases, we evaluate the empirical performance of different pHMM databases in virus identification from various metagenomic datasets. A general overview of the pipeline is depicted in Fig. 4. In the experimental design, our goal is to evaluate the raw sensitivity and specificity of using these pHMM databases on detecting viruses from heterogeneous sequencing data. No matter whether pHMM-based alignment is used as an intermediate step [5] or the main search engine [7], the raw performance of these databases can provide much needed guidance for database selection. To ensure fairness, the evaluations on all the databases follow the same pipeline and alignment parameters in Fig. 4.

Virus detection tools employing pHMMs take the assembled contigs as input. Gene prediction and translation processes are conducted first and then each derived protein sequence is aligned against pHMMs from a selected database. We utilize hmmscan module from HMMER [34] to perform comprehensive searches of each sequence against all pHMMs within the database. The contigs are then classified as viral or non-viral based on the outcomes of the hmmscan alignments.

### Performance evaluation metrics

When evaluating datasets with known labels, we assess the performance of different databases using standard metrics including recall, FP rate, and PPV of the predicted viral sequences. These metrics provide quantitative insights into the accuracy and effectiveness of the database. Recall quantifies the proportion of viral contigs accurately identified by a pHMM database out of the total number of actual viral contigs present in the dataset. The FP rate assesses the proportion of incorrectly classified non-viral contigs reported by a database relative to the total number of true non-viral contigs in the dataset. PPV calculates the percentage of true viral contigs within the set of contigs predicted to be viral by a pHMM database. For datasets lacking ground truth, we compare the outputs from using different databases with high-confidence annotation obtained using BLASTN [35]. Although this strategy cannot provide a comprehensive evaluation because BLASTN main retrieves viruses with considerable similarity with known viruses, the comparison still provides some insights into the recall and FP rate of this model.

### Filtration of pHMM and pHMM alignments

**Figure 5:**
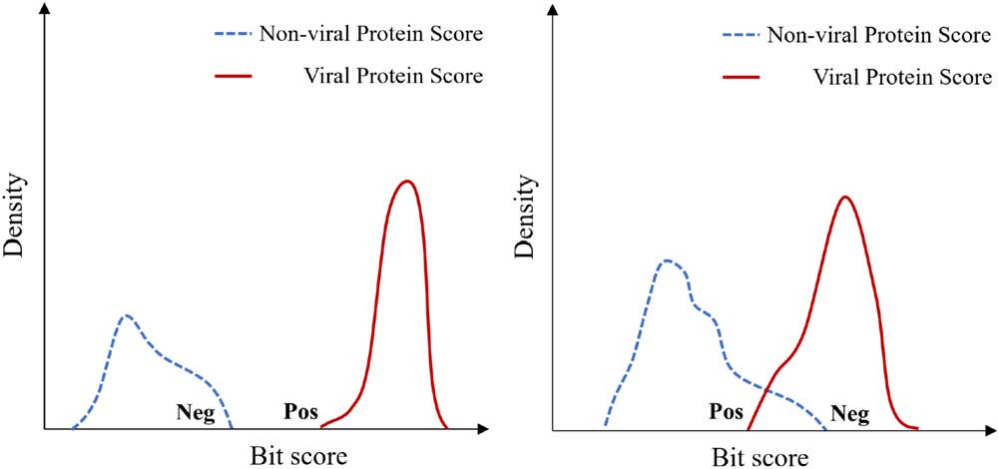
Case 3 and Case 4 for a pHMM with alignments to both viral proteins and non-viral proteins; Left: Case 3; Right: Case 4.

**Figure 6:**
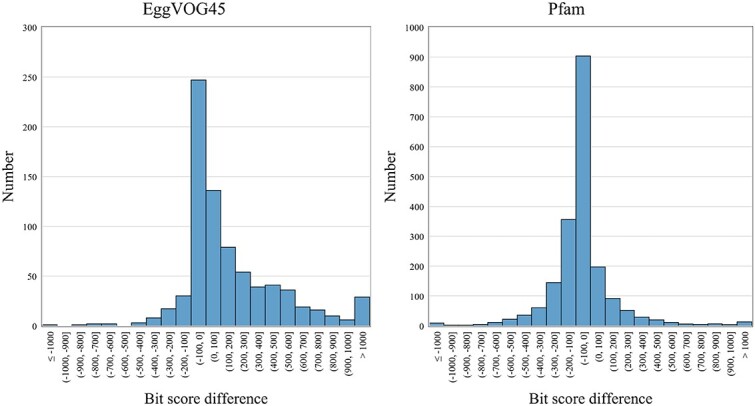
Distribution of $Pos-Neg$ for EggVOG4.5 and Pfam.

As the pHMM alignment against a sequence can produce an $E$-value and a bit-score, deciding the best cutoffs can significantly affect the virus detection performance. Although some databases provide the default score cutoffs, such as the GA cutoff by Pfam, those cutoffs may not be optimized for virus detection. Thus, besides using $E$-values as the cutoff, we implemented three model-specific score cutoffs for achieving (1) a desired tradeoff between recall and FP rate, (2) maximized recall, and (3) minimized FP rate. To determine these cutoffs, we must obtain the score distribution of each pHMM against some well annotated viral proteins and non-viral proteins. We thus downloaded reviewed proteins from UniProt. There are 15 536 viral and 555 294 non-viral proteins, respectively. Then, we run each pHMM of each reviewed database against these proteins under an $E$-value cutoff 0.01. Ideally, a useful pHMM for virus detection should be able to generate significantly higher scores for the viral proteins than the non-viral proteins. Based on the alignment results, we have four cases. In case 1, a pHMM only aligns to a viral protein and does not produce any alignment against a non-viral protein. This pHMM can be regarded as virus specific and is a good marker for virus detection. In case 2, a pHMM only aligns to a non-viral protein and does not produce any alignment against viral proteins. This pHMM is not a good choice for virus detection and thus is excluded from virus detection tasks. Case 3 and case 4 include pHMMs that can be aligned to both viral proteins and non-viral proteins. Their differences are depicted in Fig. 5. For each pHMM, denote its maximum alignment score against non-viral proteins as **Neg** and its minimum alignment score against viral proteins as **Pos**. In case 3, all the alignment scores against viral proteins are larger than non-viral proteins, resulting in a positive value for $Pos-Neg$. These pHMMs can serve as effective markers for virus detection when appropriate score cutoffs are selected. Conversely, in case 4, the scores against viral and non-viral proteins overlap, yielding a negative value for $Pos-Neg$. Despite the fact that most of the reviewed pHMM databases are constructed using viral proteins, this analysis demonstrates that they can still generate alignments against non-viral proteins. The numbers of models in each case for every database are provided in Supplementary Fig. S9. Case 3 and case 4 are quite common, meaning that many viral proteins are homologous with non-viral proteins. Figure 6 shows the distribution of $Pos-Neg$ for EggVOG4.5 and Pfam. The score distribution of all 7 pHMM databases can be found in Supplementary Fig. S5. As the figure shows, many viral protein pHMM databases can incur hits against non-viral proteins. Without keeping this in mind, users may report many false positive viral contigs.

To optimize the usage of pHMMs for virus detection, we thus propose three model-specific cutoffs based on the score distribution. The first cutoff is similar to the machine learning model Support Vector Machine, which chooses the decision boundary to maximize the margin with the negative and positive samples. Here, we use the middle point between Pos and Neg as the first cutoff named $T_{mid}$, which is calculated as $1/2 \times (Pos+Neg)$. The second cutoff, named as $T_{max{\_ }recall}$, is computed for case 3 and case 4 using different equations. To maximize the recall, $T_{max{\_ }recall}=Neg$ for case 3 and $T_{max{\_ }recall}=Pos$ for case 4. The third cutoff, named as $T_{min{\_ }FP}$, is used to minimize the FP rate. Thus, $T_{min{\_ }FP}=Pos$ for case 3 and $T_{min{\_ }FP}=Neg$ for case 4.

**Additional filtration for Pfam and KOfam** For all the databases that are constructed using viral proteins, we classify a contig as virus as long as one of the predicted ORF is classified as virus protein.

Because Pfam is a comprehensive protein family/domain database, we classified the models into two groups based on the proportion of viral sequences in each model’s training data. Group A consists of non-viral models, and Group B includes models where the viral ratio surpasses a certain predefined threshold (10% by default). Group A contains 14 955 models, none incorporating any viral sequences. This group represents approximately three-quarters of all models within Pfam. Of all the 1860 models in Group B, 191 contain only viral proteins, and 68% of Group B models contain at least 80% viral proteins. As one contig can incur matches against multiple pHMMs, a feasible strategy to distinguish viral contig from non-viral ones is to stipulate that the count of alignments to Group B (viral models) must surpass that to Group A (non-viral models). In essence, sequences are deemed as potential viral candidates only if they exhibit a greater number of hits to viral models compared with non-viral ones.

As a subset of KOfam models are used by CheckV as markers for viruses, we filter hits only to include alignments from these 78 identified viral models in all downstream analyses for viral sequence prediction.

## Experimental results

We evaluated the performance of pHMMs across three datasets exhibiting progressively increasing complexity. The first dataset, generated using CAMISIM, consists of 34 viral contigs and 173 921 bacterial and fungal contigs. Predominantly composed of RNA viruses, all viruses in this synthetic dataset are capable of infecting human cells. The ground truth labels for each contig enable precise calculation of recall, FP rate, and PPV. The second dataset, a semi-simulated environmental sample, was obtained from the NCBI Sequence Read Archive (SRA). Among its 29 570 contigs, there are 202 identified as viral, with RNA viruses constituting only 10%. The third dataset is derived from an actual metagenomic sample collected from a pilot-scale staged anaerobic fluidized membrane bioreactor, comprising 312 399 contigs with an unknown viral composition. The latter two datasets mainly contain prokaryotic viruses, reflective of their metagenomic origins. Given the incomplete annotation of many prokaryotic viral genomes, their associated proteins may be underrepresented in existing pHMM databases. This presents a challenging yet critical test for the comprehensive evaluation of pHMM databases in real-world scenarios.

### Experiment 1: simulated metagenomic dataset

In this experiment, we generated a metagenomic dataset using CAMISIM [36]. This dataset comprises more than 30 different types of bacteria, fungi, and over 10 types of viruses. The detailed composition can be found in Supplementary Table S1. CAMISIM generated both sequencing reads and simulated assembled contigs. The taxonomic labels for contigs are known, with 34 viral contigs among 173 955 contigs. Based on these ground truth labels, we compared the virus detection performance of using seven different databases in this simulated dataset. As each pHMM alignment has associated $E$-value and score, we present the performance by using both $E$-value and model-specific score cutoffs.

#### Results of using different cutoffs

The section presents the results of using a strict $E$-value cutoff (0.0001), a lenient $E$-value cutoff (10), and three model-specific score cutoffs.

**Figure 7:**
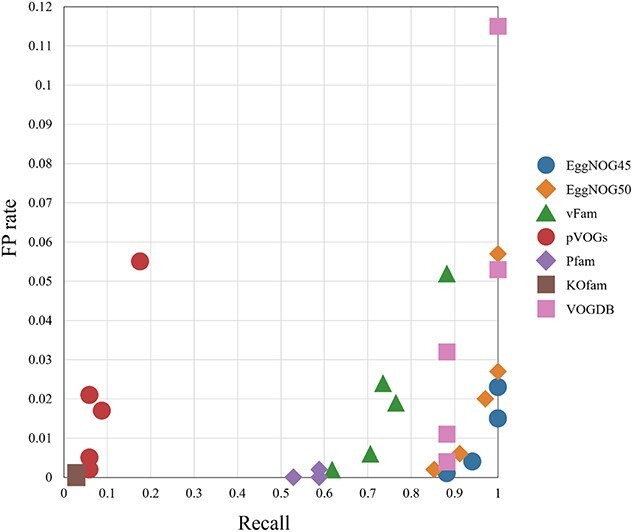
The scatter plot of recall and FP rate for 7 pHMM databases’ virus detection results; all the metrics are evaluated on contigs, and each database has 5 dots, corresponding to the results of two $E$-value cutoffs and three model-specific cutoffs; Figure 8 shows the performance for each cutoff.

Figure 7 shows the scatter plot of FP rate and recall of using 7 pHMM databases for viral contig detection. The main purpose is to examine which database leads to better results in this experiment. Two EggNOG databases exhibit clustering of dots at the lower right corner, indicating high recall with a low FP rate. VOGDB achieved high recall, but the best FP rate is still higher than EggNOG. Pfam always has a low FP rate because of our additional filtration step. Only using $T_{min{\_ }FP}$ leads to 883 hits with 861 of them being FP hits. By using the additional filtration step as described in Section “Additional filtration for Pfam and KOfam,” the total hits is reduced to 21 with only 3 as being FP hits. Thus, for a comprehensive database such as Pfam, additional filtration is still needed to achieve a low FP rate.

**Figure 8:**
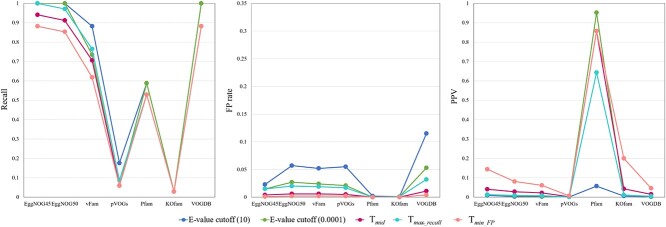
$X$ -axis: pHMM database; $Y$-axis: performance metrics; different cutoffs affect FP and PPV but have minimal impact on recall in this experiment; note that some of the databases yield the same values for recall or PPV, resulting in a single clustered dot in the visualization; for example, the recall for VOGDB is 0.882 under three $E$-value cutoffs, $T_{mid}$, $T_{max{\_ }recall}$, and $T_{min{\_ }FP}$, resulting in a single clustered dot; a similar pattern is observed with EggNOG4.5 and EggNOG5.0, and Pfam has nearly identical recalls under two $E$-value cutoffs, and the PPV for Pfam is 0.857 under two $E$-value cutoffs, $T_{mid}$, and $T_{min{\_ }FP}$, resulting in a single clustered dot; KOfam has the same recall under all cutoffs.

Figure 8 shows the recall, FP rate, and PPV of using 7 pHMM databases for virus search under five different cutoffs. The results are consistent with the design purpose of the cutoffs. Using $T_{max{\_ }recall}$ and $T_{min{\_ }FP}$ leads to the maximum recall and minimum FP for all. By comparing different cutoffs, using $T_{min{\_ }FP}$ has the lowest FP and reasonable recall. In addition, it leads to the highest PPV for all databases except Pfam. The database pVOG does not achieve high recall because this dataset mainly contains RNA viruses, while pVOG contains prokaryotic viruses. Pfam has the highest PPV by using a stringent $E$-value cutoff. We also tested its performance by using the default GA cutoff. Its recall, FP rate, and PPV under the GA cutoff are 0.971, 0.001, and 0.202, respetively. There is no clear advantage of using GA cutoff in this experiment because of the low PPV value. black

**Results of using $E$-value cutoffs and model coverage cutoffs** We further tested and recorded the performance of different pHMM databases by adjusting $E$-value cutoffs. The detailed results can be found in Supplementary Fig. S6 and Supplementary Table S2. Overall, using $E$-value as cutoffs yields worse performance (smaller PPV) than model-specific cutoffs.

We then investigate whether we can reduce the FP rate using the alignment coverage on the pHMM models. In this analysis, we fix the $E$-value threshold at a stringent value ($1.0E-03$), and then, we increase the threshold for the model coverage from 0 to 1 with the step size of 0.2. The detailed results can be found in Supplementary Table S3.

### Experiment 2: semi-simulated dataset

In this section, we assess the pHMM databases on a semi-simulated metagenomic dataset [37] (NCBI SRA accession number: ERR1992810). This dataset comprises reads obtained from 82 eukaryotes, 365 prokaryotes, and 87 DNA/RNA viruses, simulating real marine water samples with sequencing error profiles derived from real data. The reads were then assembled using MEGAHIT [38]. There are many short contigs with N50 as 720. Because the composition is provided, we obtained the taxonomic labels of the contigs by aligning them against the reference genomes using BLASTN.

#### Results of using BLAST labeled ground truth

For this semi-simulated dataset, we assign labels of the contigs by running BLASTN against all known viral genomes provided by the dataset composition and the NCBI nt database. Among contigs that have alignments against the reference genomes, sequences that can be aligned to known viral genomes with high identities (over 70%) are labeled as viral sequences, the rest of the sequences are labeled as non-viral sequences. Contigs that have no alignment with any sequences from the NCBI nt database are labeled as unknown sequences. These three labeled sets serve as our ground truth for evaluation. Out of 29 570 total contigs with length above 1000bp, 202 contigs were labeled as viral contigs, 21 091 contigs were labeled as non-viral contigs, 8277 contigs were labeled as unknown contigs.

Based on the results of the first experiment, we tested the virus detection performance of all databases under two stringent $E$-value cutoffs 0.001, 0.0001 and the three model-specific score cutoffs. Each pHMM database’s predicted viral sequences are compared against three BLAST-labeled sets. The classification of predicted viral sequences is based on their agreement with the BLAST labels. If a predicted viral sequence is labeled as viral by BLAST, it is classified as a true positive. Conversely, if it is labeled as non-viral, it is considered a false positive. Predicted sequences that are labeled as unknown by BLAST remain categorized as unknown.

**Figure 9:**
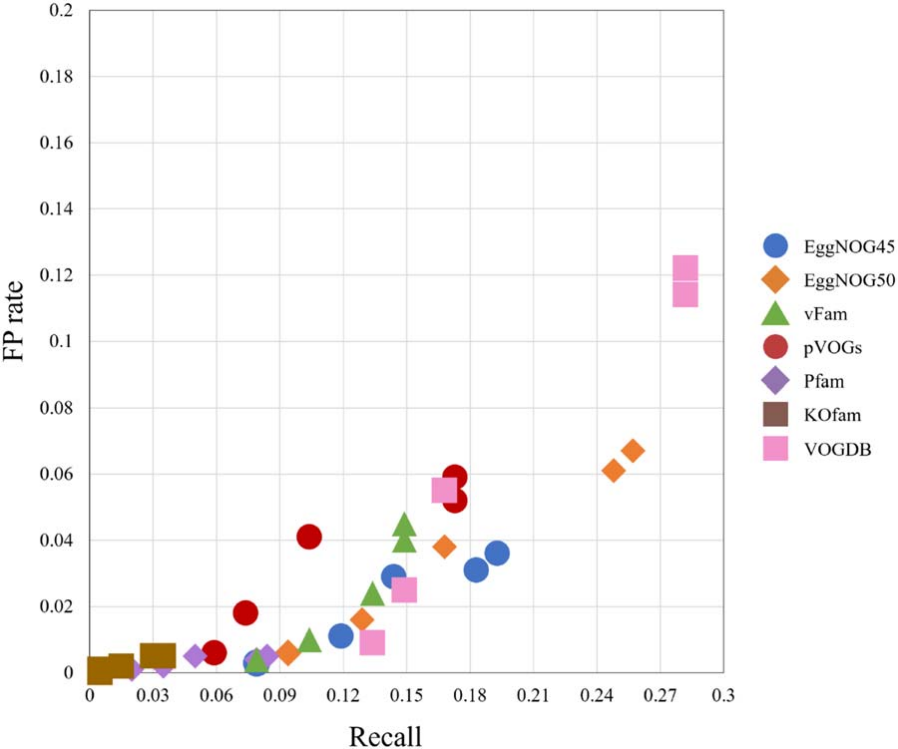
The scatter plot of recall and FP rate for 7 pHMM databases’ virus detection results; all the metrics are evaluated on contigs; each database has 5 dots, corresponding to the results of two $E$-value cutoffs and three model-specific cutoffs; Figure 10 provides mapping between the performance and the 5 cutoffs.

**Figure 10:**
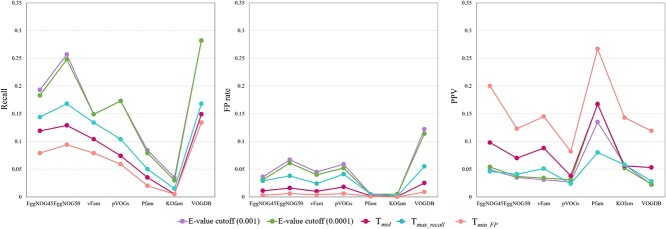
$X$ -axis: pHMM database; $Y$-axis: performance metrics.

This dataset poses challenges for all pHMM databases. The recall-FP scatter plot in Fig. 9 shows that the increase of recall is often accompanied by the increase of FP rate. No database shows a clear advantage. Different from the first dataset, most of the viruses here are prokaryotic viruses. Thus, pVOGs has improved recall compared with the first experiment. Using PPV as the main metric, the score cutoff $T_{min{\_ }FP}$ yields the best PPVs for various databases, according to Fig. 10.

#### Investigation of low recall

In comparison to the first dataset, all pHMM databases exhibit low recall for viral contig detection within the second dataset. VOGDB demonstrates the highest recall, achieving 0.282 at the selected $E$-value cutoff, indicating that roughly 80% of the 202 viral sequences identified by BLAST remain undetected by most databases.

The limited detection is primarily due to the greater viral diversity present in the second dataset, which comprises viruses in environmental samples, including $\sim $10% RNA viruses and a predominance of DNA viruses. This diversity contrasts with the first dataset, which contains only human-infecting viruses, predominantly human RNA viruses.

Environmental samples generally harbor a broader spectrum of viruses compared with human samples. Human viral pathogens are more thoroughly researched, and their proteins are well-characterized within existing databases. In contrast, bacteriophages, which constitute a significant portion of DNA viruses in environmental samples, often have incompletely annotated sequences, and many phage proteins are underrepresented in current databases. Consequently, existing pHMM databases lack comprehensive coverage of protein families or domains found in these diverse viral populations.

**Figure 11:**
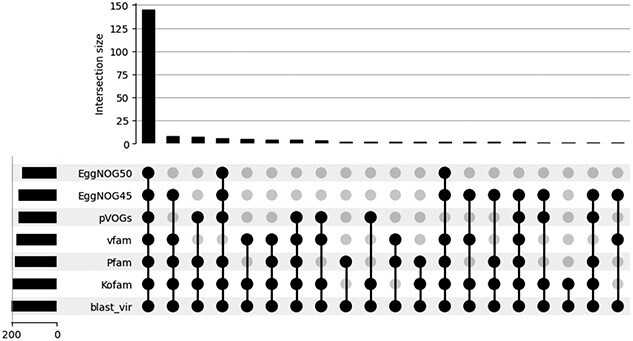
Upset plot of the FN from different pHMM databases on the semi-simulated dataset; $X$-axis: combination of the databases (black dots); $Y$-axis: the number of FN contigs commonly reported by this combination; for example, there are more than 140 sequences that are missed by all six databases (six black dots); the BLAST-annotated viral sequences are used of reference here.

Many of these missed viral contigs are common to all the databases. Figure 11 presents an upset plot illustrating the false negative sequences that are missed by various databases under the $E$-value cutoff 0.0001. Notably, nearly 150 sequences are missed by all databases.

To gain further insights, we extract the five longest sequences from this common false negative (FN) set and analyze the alignment regions of these sequences in the BLAST results. The average length of the five sequences is 3531 bases. We use the six-frame translation for finding ORFs. These five sequences all contain very long ORF in one reading frame. They belong to different types of viruses. The most likely reason is that their proteins are not included in the current versions of the pHMM databases. For example, the longest viral contig is from the genome of Vibrio phage PG216. It can be aligned with a more recent Pfam model PF21427 (Baseplate wedge protein gp7, domain V) when we conducted an online HMMER search. However, this Pfam model is not present in the version we reviewed (Pfam 35.0, last updated on 3 January 2023).

**Control FP rates with hit coverage** To control the high FP rate, we also tested whether using both the alignment coverage on the model and on the contig can further reduce the FP rate. The detailed results can be found in Supplementary Table S4.

### Experiment 3: real metagenomic dataset

In this section, we extend our evaluation of the performance of pHMM databases on a real sample. The bio-sample was obtained from a pilot-scale staged anaerobic fluidised membrane bioreactor (SAF-MBR) at the Resource Recovery Center, Stanford University, California [39]. This SAF-MBR, which has been treating municipal wastewater from the Serra Street sewer on the university campus for over 3 years, provides a realistic representation of real-world conditions. The system employs granular activated carbon as a scouring agent in the membrane system, which also serves as a biocarrier to promote microbial growth and biodegradation by offering a large surface area for biofilm formation. The GAC samples were collected after the reactor reached a steady state. The biomass was harvested from the GAC using low-power sonication in an ice bath. DNA were extracted using the DNeasy PowerSoil kit and RNeasy Kit (Qiagen, Hilden, Germany), respectively, and stored in 50 % ethanol solution (Thermo Fisher, MS, USA).

The samples underwent shotgun metagenomic using Illumina NovaSeq (Illumina Inc, San Diego, CA, USA), generating 20 Gb of metagenome sequences. The DNA sequences were denoised using Cutadapt v4.0 [40] and Trimmomatic v0.39 [41]. The metagenome was assembled using MEGAHIT v1.2.9 [38], and gene calling for the assembled contigs was performed using Prodigal-GV v2.11.0 [42], preparing the sequences as HMMER inputs.

#### Adjusting BLAST labels

In this dataset, the taxonomic labels for both the reads and contigs remain unknown. We employ BLASTN against the NCBI nt database to assign labels to the contigs. Supplementary Figure S7 depicts the Venn diagram illustrating the overlap between the aligned contigs and the entire set of 312 399 contigs. Notably, the diagram reveals that over 40% of the contigs fall into the category of “dark matter.”

To mitigate the potential impact of mislabeling, we take great care in curating the criteria for BLAST labeling. Specifically, we consider both the percentage identity (pid) and the query sequence coverage (cov) of the BLAST alignment. We only classify sequences as viral if their pid*cov value against a virus genome exceeds a certain threshold. For a pid*cov cutoff set at 0.25 (0.5 * 0.5), we label 392 contigs as viral sequences. When the pid*cov cutoff is raised to 0.4 (0.8 * 0.5), 305 contigs are designated as viral sequences. Further increasing the pid*cov cutoff to 0.64 (0.8 * 0.8) results in 206 contigs being labeled as viral sequences.

#### Results under different BLAST labels

Without knowing the labels of most contigs, we run different databases under the stringent $E$-value cutoff $1.0E-04$ and the model-specific score cutoff $T_{min{\_ }FP}$. As this sample contains a large number of contigs, even a small FP value can lead to a significant number of non-viral contigs. Thus, we only use the score cutoff designed to minimize the FP rate.

For this sample, only three pHMM databases (EggNOG 4.5, EggNOG 5.0, and pVOGs) yield discernible results under these two different cutoffs. The two comprehensive databases, Pfam and KOfam, do not predict viral sequences after applying the model filtering.

To provide a quantitative analysis, Table 2 examines the recall rates of the databases. Notably, pVOGs exhibits the highest recall, reaching 0.574 under the most relaxed BLAST labeling criteria. PVOG is a pHMM database for prokaryotic viruses. Considering that this sample mainly contains prokaryotic viruses, this partially explains why pVOGs yields higher recall. With our model-specific cutoff $T_{min{\_ }FP}$, the returned contigs are significantly reduced, which is expected. The recall is also reduced. But for users who wish for a more reliable prediction, using this cutoff can lead to smaller FP rate.

**Table 2:** Results with different BLAST labeling strategies; two cutoffs are used: $E$-value cutoff $1.0E-04$ and the model-specific cutoff $T_{min{\_ }FP}$

**Cutoff**	**label**		Egg4.5	Egg5.0	pVOGs
$E$ -value $1.0E-04$		predicted	52 087	111 963	99 691
	>0.64(206)	**TP**	17	97	105
		**Recall**	0.0825	0.471	0.510
	>0.4(305)	**TP**	30	145	169
		**Recall**	0.098	0.475	0.554
	>0.25(392)	**TP**	42	193	225
		**Recall**	0.107	0.492	0.574
Cutoff: $T_{min{\_ }FP}$		predicted	4419	10 312	10 264
	>0.64(206)	**TP**	4	20	23
		**Recall**	0.019	0.097	0.112
	>0.4(305)	**TP**	5	30	44
		**Recall**	0.016	0.098	0.144
	>0.25(392)	**TP**	8	46	70
		**Recall**	0.02	0.117	0.179

## Conclusion and discussion

This review covers the intrinsic properties of commonly used pHMM databases that cover viruses. As each pHMM is constructed using an MSA and a similar model construction algorithm, the main differences of various pHMM databases stem from the seed sequences used to build the model and how these sequences are clustered into different protein domains or families. We thus first compared the basic statistics of these databases, including the number of models, the training data (virus or comprehensive), model length, and the number of training sequences for each model. To reflect the resolution of the pHMMs, meaning the granularity of the clustering process, we introduced an entropy-based method to evaluate the models’ diversity at each taxonomic rank. A small entropy value at a taxonomic rank indicates that the model is more homogeneous for this taxa group. For users who need to use pHMMs as markers of specific taxonomic groups, this information is particularly useful.

Furthermore, we evaluated the performance of different pHMM databases in detecting viral sequences across simulated, semi-simulated, and real-life metagenomes. The three datasets increase in complexity and scale. To optimize the performance of virus detection, we designed model-specific score cutoffs, which exhibit better performance than using the same $E$-value cutoff for all pHMMs. Based on our findings, we would like to highlight several key considerations for users.

First, users should take great caution in using the viral pHMM databases. Although these databases are built using viral proteins, this does not guarantee that they would not be aligned to non-viral proteins. Our analysis shows that many of these pHMMs can incur statistically significant alignments against non-viral proteins. Overall, our analysis shows that it is very rare that a pHMM only aligns to viral proteins, indicating that viral proteins are often homologous to some non-viral proteins. Thus, using model-specific cutoff can help reduce FP.

Second, current pHMM databases yield much better performance in identifying RNA viruses (Exp 1) than prokaryotic viruses (Exp 2 and 3). Most RNA viral proteins have been covered by available protein families/domains. In contrast, most prokaryotic viruses do not have marker proteins. Their high diversity and under-studied annotation contribute to the dark matter of viruses. In the third experiment, pVOGs has better recall than others because it mainly covers prokaryotic viruses. Considering that the annotation of proteins from newly sequenced falls behind the fast accumulation of the sequences, using BLASTN-based pairwise alignment can help improve the recall. With the increased coverage of the pHMMs for prokaryotic protein domains, this issue will be mitigated. But now it is one limitation of pHMM-based virus detection. Meanwhile, other deep learning-based tools that use features beyond homology search have reported better recall for identifying prokaryotic viruses [43].

Third, it can be observed that comprehensive databases including Pfam and KOfam incur the lowest FP rate in virus search because they can leverage pHMMs for non-viral proteins to remove non-viral contigs. Thus, for users who are highly cautious about false positives should use the comprehensive databases.

While our evaluation of multiple pHMM databases is extensive, it is constrained by the protein data utilized from UniProt. Due to the substantial computational demands of aligning all pHMMs from seven databases against UniProt’s proteins, we have opted to establish score cutoffs using only reviewed proteins. This approach can be expanded to encompass both reviewed and unreviewed proteins, the latter of which includes many from prokaryotic viruses, as computational resources increase. An additional limitation of our study is the exclusion of certain pHMM databases that are not as commonly used but employed by specific research groups. Our focus was on those databases that are widely recognized and utilized; however, our methodology is adaptable and could be applied to a more comprehensive set of pHMM databases. A further challenge we faced in comparing different pHMM databases was the absence of a definitive ground truth for complex real-world metagenomic datasets. Consequently, we had to depend on BLAST to provide labels, which offers a very limited scope of reference.

**Key Points**

- New and highly diverged viruses comprise viral dark matter in (viral) metagenomic data. Profile hidden Markov model-based search is a key step in virus discovery and characterization. Given many different pHMM databases, there lack a comprehensive review about choosing the proper databases for virus search. This review offers both data analysis and empirical evaluations of multiple commonly used pHMM databases for virus discovery.
- We reviewed eight commonly used pHMM databases by comparing their sizes, their taxonomic coverage, and the properties of their models using quantitative metrics. We devised an entropy-based metric to evaluate the conservation or purity of a pHMM. By applying this metric, we obtained taxonomic conservation for various pHMM databases, offering valuable insights to users in the database selection process.
- We examined all pHMMs’ alignment scores against viral proteins and non-viral proteins. Then, we designed three types of model-specific cutoffs. We conducted a series of experiments to assess the virus detection performance with different pHMM databases, which can provide users important practical guidance.

## Supplementary Material

supp_bbae292

5FN-protein_bbae292

## Author Biographies

**Runzhou Yu** completed her PhD in the Department of Electrical Engineering at City University of Hong Kong. Her research interest includes sequence analysis in bioinformatics, with a focus on algorithm design for analyzing sequencing data.

**Ziyi Huang** is currently research assistant in the Department of Electrical Engineering at City University of Hong Kong. His research interests include bioinformatics and machine learning.

**Theo Y.C. Lam** was a research assistant in the Department of Electrical Engineering at City University of Hong Kong. He completed his PhD in Environmental Engineering in the Department of Civil and Environmental Engineering at Imperial College London. His research interests include wastewater treatment, metagenomics, bioinformatics and microbiome.

**Yanni Sun** is a professor in the Department of Electrical Engineering at City University of Hong Kong. Before she relocated to Hong Kong, she was an Associate Professor in the Department of Computer Science and Engineering at Michigan State University, USA. She received the BS and MS degrees from Xi’an Jiao Tong University (China), both in Computer Science. She received the PhD degree in Computer Science and Engineering from Washington University in Saint Louis, USA. She works in bioinformatics and computational biology. In particular, her recent research interests include sequence analysis, application of machine learning and data mining models in analyzing nextgeneration sequencing data, metagenomics, protein domain annotation, and noncoding RNA annotation. She was a recipient of NSF CAREER Award in 2010.
