# Supplementary material for: Utilizing profile hidden Markov model databases for discovering viruses from metagenomic data: a comprehensive review
Source: Brief Bioinform. 2024 Jun 20;25(4):bbae292. doi: 10.1093/bib/bbae292 (PMC11246558; doi:10.1093/bib/bbae292)
Supplement: supp_bbae292 [file supp_bbae292.pdf]

Utilizing profile HMM databases for discovering viruses from metagenomic data: a comprehensive review Supplementrary Material

Runzhou Yu, Ziyi Huang, Theo Y.C. Lam, and Yanni Sun

May 19, 2024

1 Supplementary Figures

List of Figures

S1 Distribution of model lengths and training sequences across seven pHMM databases. . . 2
S2 Taxonomic tree of the RNA Helicase model (PF00910) in Pfam. 3
S3 Raw Entropy value distribution of four pHMM databases. 4
S4 Entropy values at all taxonomic ranks for 10 largest models from vFam and Pfam. 4
S5 The distribution of Pos-Neg for the pHMM databases. 5
S6 Virus detection results by adjusting the E-value cut-off in Exp 1. 6
S7 Venn diagram between the BLAST-aligned contigs and all contigs. 6
S8 Entropy value distribution for viral only pHMMs (group B) in Pfam. 7
S9 Model number distribution for 4 cases. 7

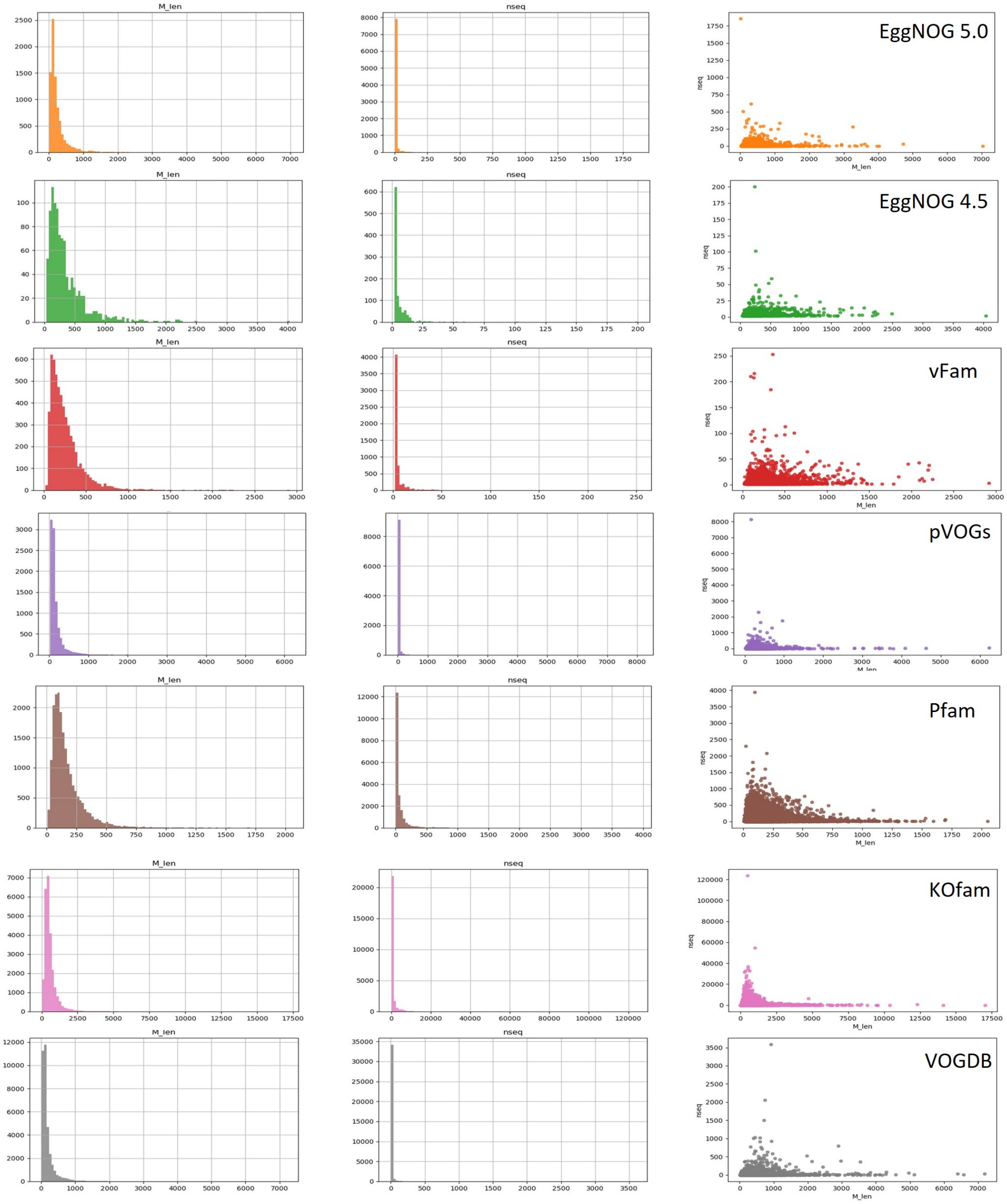

Figure S1: The distribution of model lengths and training sequences across seven pHMM databases, listed from top to bottom: EggNOG 5.0, EggNOG 4.5, vFam, pVOGs, Pfam, KOfam, and VOGDB. Left: distribution of model lengths; Middle: distribution of number of training sequences; Right: scatter plot of model lengths and the corresponding number of training sequences.

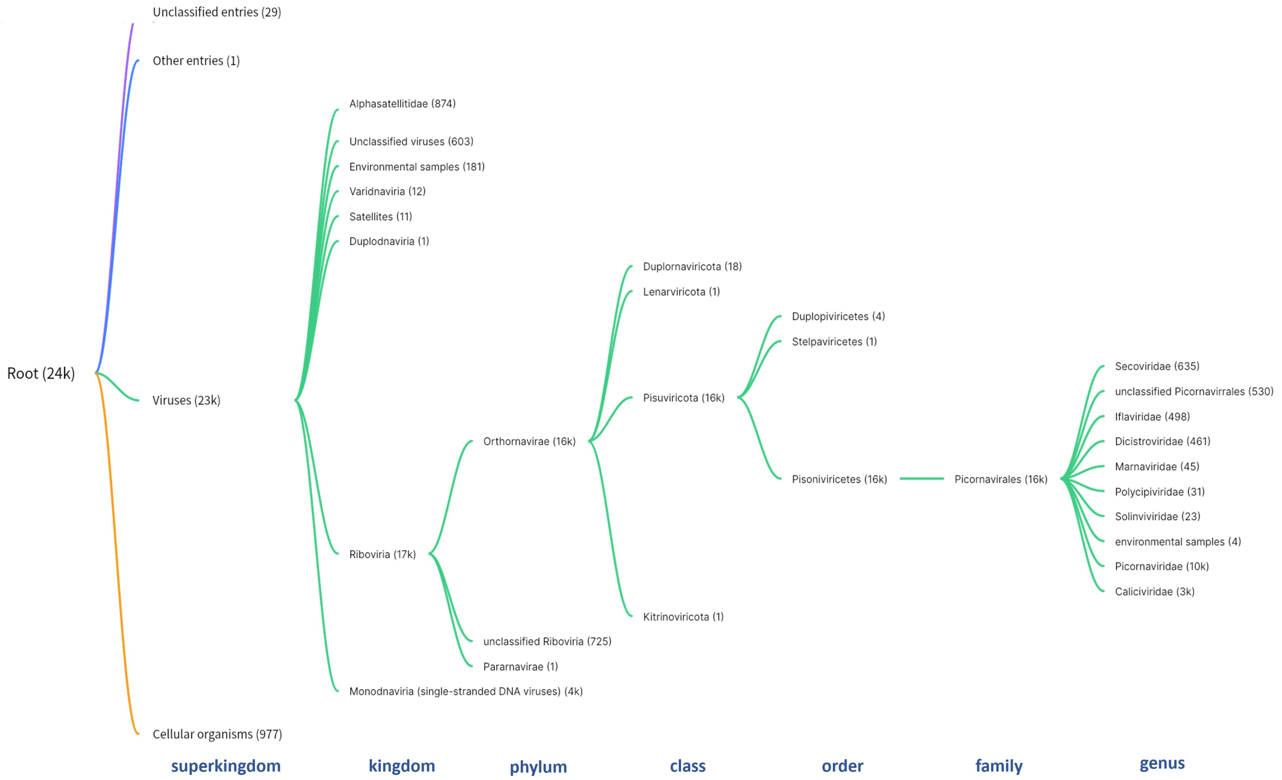

Figure S2: The taxonomic tree from superkingdom level to genus level composed of the training sequences of the RNA Helicase model (PF00910) in Pfam. Only the dominant branch (the branch with the greatest number of training sequences) is shown in this example.

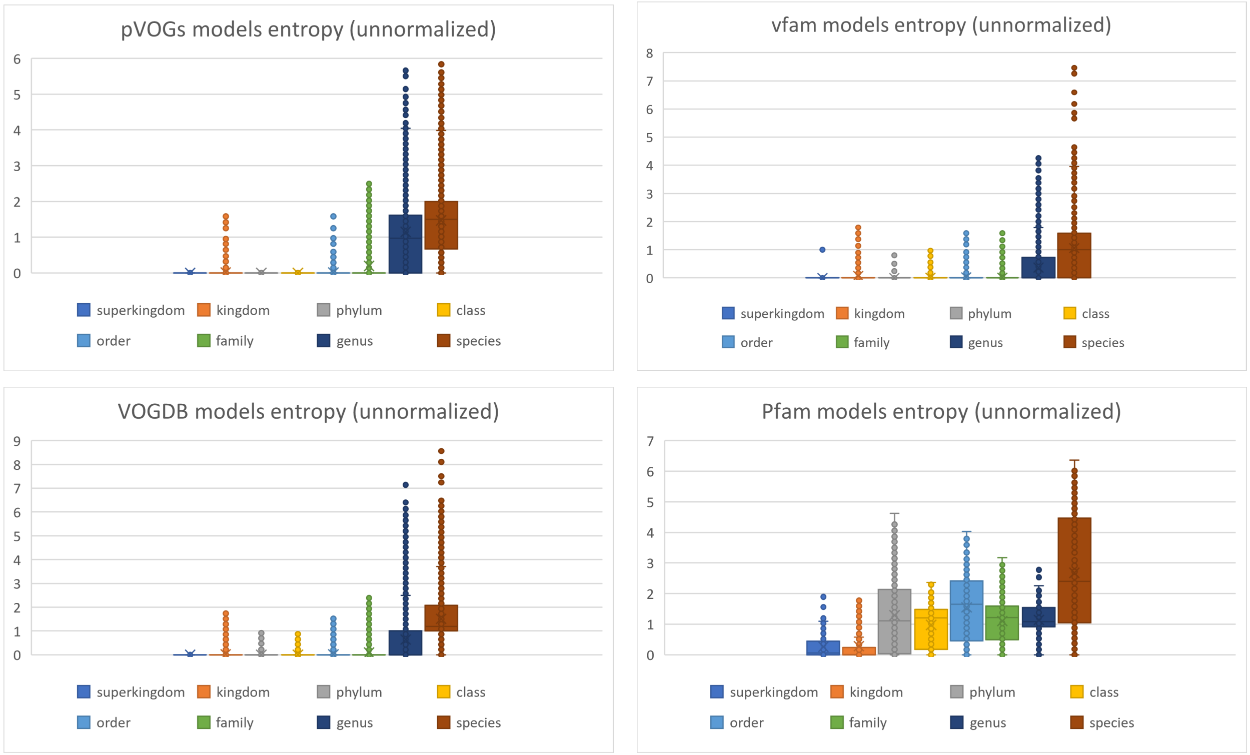

Figure S3: Raw Entropy value distribution of four pHMM databases. As these are unnormalized Shannon Entopy values, the max values and y-axis ranges for each figure are different.

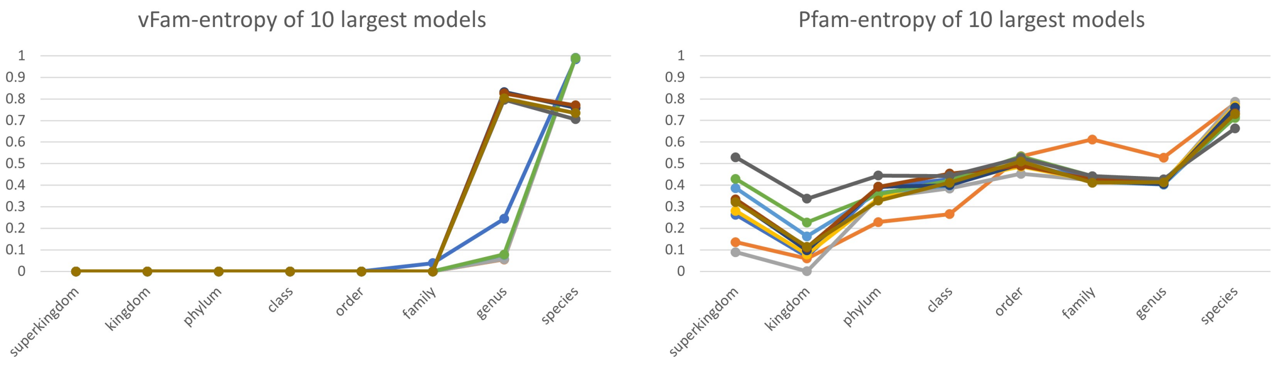

(a) (b)

Figure S4: Entropy values at all taxonomic ranks for 10 largest models from (a) vFam and (b) Pfam.

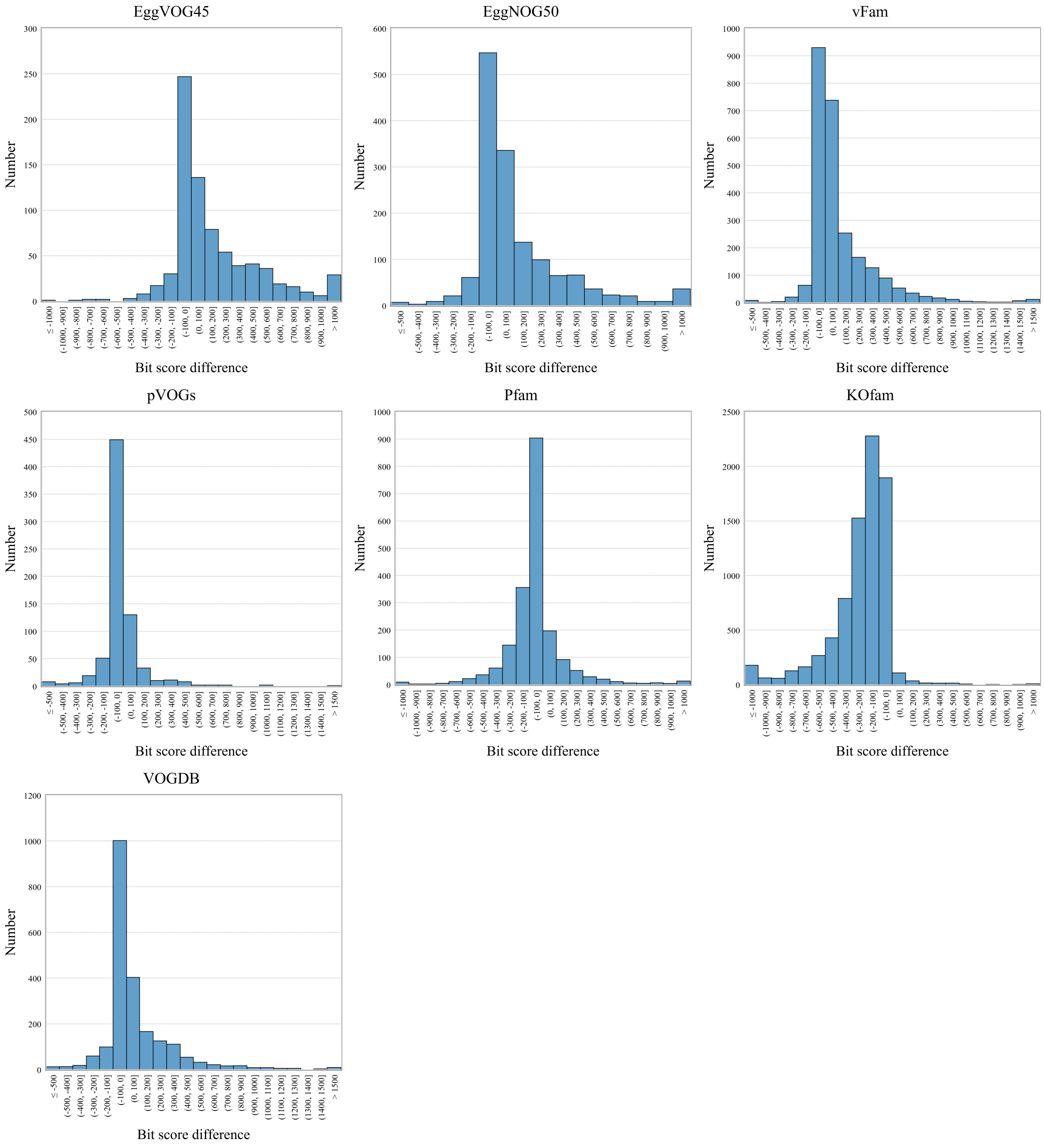

Figure S5: The distribution of $Pos - Neg$ for the pHMM databases. Only pHMMs that can be aligned to both viral proteins and non-viral proteins are included here.

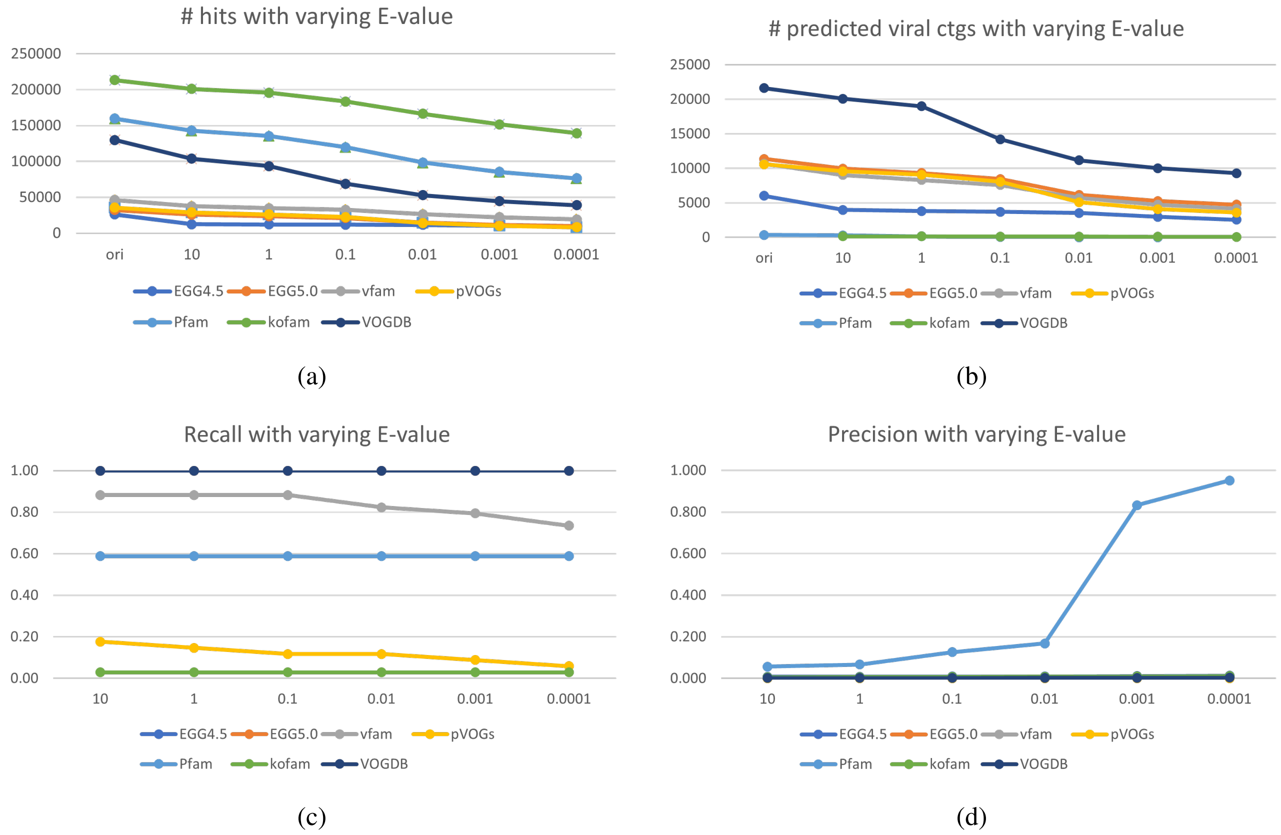

Figure S6: Virus detection results by adjusting the E-value cut-off on the simulated metagenomic dataset. (c) The recall curves for EGG4.5 and EGG5.0 overlap with VOGDB. (d): The precision values of all databases except Pfam have similar trend and thus largely overlap in this figure.

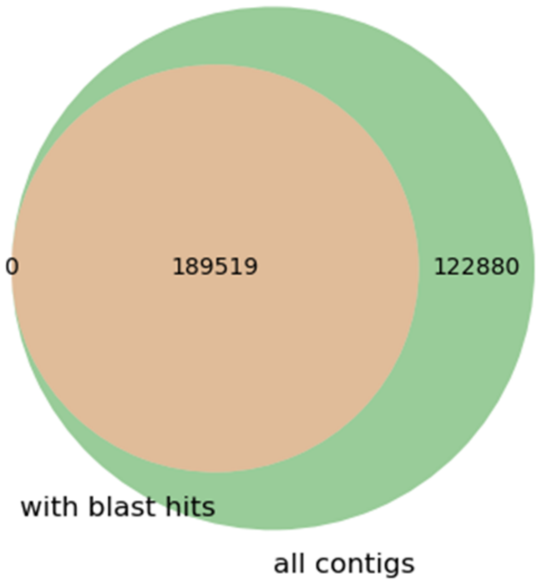

Figure S7: Venn diagram between the BLAST-aligned contigs and all contigs.

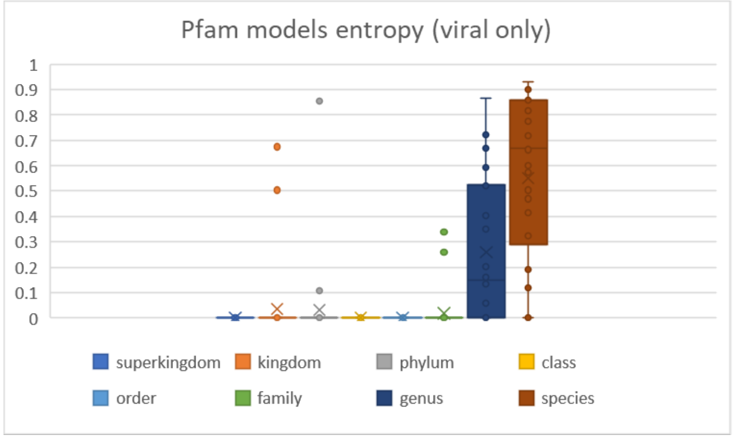

Figure S8: Entropy value distribution for viral only pHMMs (group B) in Pfam.

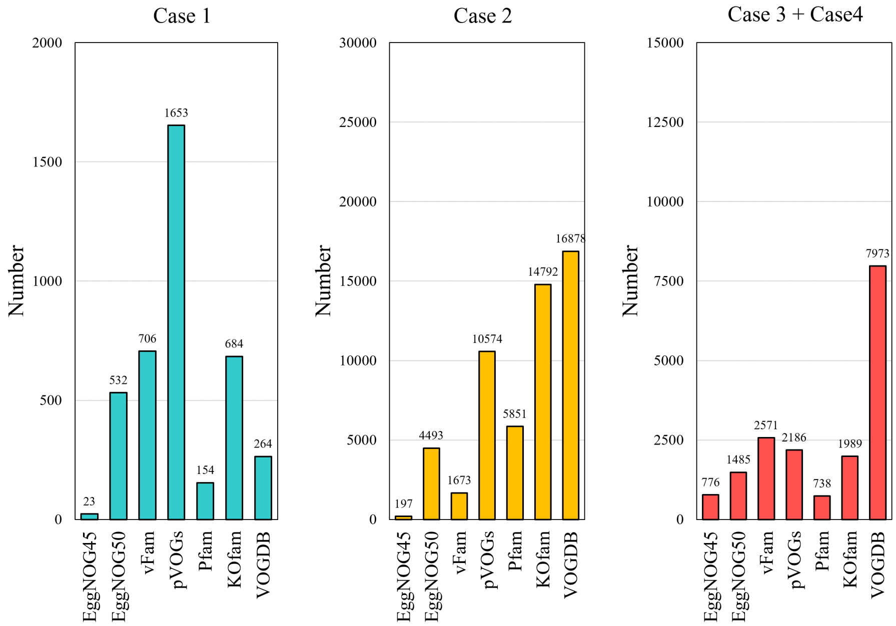

Figure S9: The number of models for 4 cases in each database. X-axis: pHMM database. Y-axis: numbers for 4 cases. Case 1: The number of pHMMs that only align with viral proteins; Case 2: The number of pHMMs that only aligns with non-viral proteins; Case 3 and Case 4: The number of pHMMs that can be aligned to both viral proteins and non-viral proteins.

2 Supplementary Tables

List of Tables

S1 Taxonomic composition profile of the simulated metagenomic dataset. 9
S2 Virus detection performance by adjusting the E-value cut-off on the simulated metagenomic dataset. 10
S3 Virus detection performance by adjusting the model coverage cut-off on the simulated metagenomic dataset. 10
S4 Prediction results for different coverage cut-off combinations. 11

Table S1: Taxonomic composition profile of the simulated metagenomic dataset.

**Taxonomic ID**	**Type**	**Name**	**Reads Fraction**
28137	Bacteria	Prevotella veroralis	0.0813
287	Bacteria	Pseudomonas aeruginosa	0.5737
28124	Bacteria	Porphyromonas endodontalis	0.2108
1313	Bacteria	Streptococcus pneumoniae	1.0069
83558	Bacteria	Chlamydia pneumoniae	0.0713
485	Bacteria	Neisseria gonorrhoeae	8.4703
28134	Bacteria	Hoylesella oralis	0.6302
111015	Bacteria	Actinomyces radicidentis	0.5723
1773	Bacteria	Mycobacterium tuberculosis	0.8014
28123	Bacteria	Porphyromonas asaccharolytica	1.9425
727	Bacteria	Haemophilus influenzae	0.4898
2104	Bacteria	Mycoplasmoides pneumoniae	0.0056
29466	Bacteria	Veillonella parvula	0.2146
1314	Bacteria	Streptococcus pyogenes	0.0757
837	Bacteria	Porphyromonas gingivalis	0.4093
1658	Bacteria	Actinomyces bovis	0.966
1317	Bacteria	Streptococcus downei	0.1099
1308	Bacteria	Streptococcus thermophilus	0.7228
1280	Bacteria	Staphylococcus aureus	0.1957
28132	Bacteria	Prevotella melaninogenica	0.2123
1334	Bacteria	Streptococcus dysgalactiae	0.1303
573	Bacteria	Klebsiella pneumoniae	23.3267
446	Bacteria	Legionella pneumophila	0.2173
2047	Bacteria	Rothia dentocariosa	0.1504
1655	Bacteria	Actinomyces naeslundii	0.1807
40542	Bacteria	Leptotrichia buccalis	0.1553
143361	Bacteria	Filifactor alocis	0.9664
194197	Bacteria	Neisseria dentiae	0.2868
37923	Bacteria	Rothia kristinae	0.6873
5059	Eukaryota	Aspergillus flavus	1.0712
5482	Eukaryota	Candida tropicalis	0.0803
746128	Eukaryota	Aspergillus fumigatus	0.0801
42068	Eukaryota	Pneumocystis jirovecii	1.2185
563466	Eukaryota	Scedosporium apiospermum	1.838
5476	Eukaryota	Candida albicans	1.4694
5480	Eukaryota	Candida parapsilosis	0.3788
11320	Viruses	Influenza A virus	24.6286
129951	Viruses	Human mastadenovirus C	0.7047
11137	Viruses	Human coronavirus 229E	4.9163
694009	Viruses	Severe acute respiratory syndrome-related coronavirus	0.2143
290028	Viruses	Human coronavirus HKU1	0.6468
11250	Viruses	Human orthopneumovirus	0.6619
147712	Viruses	Rhinovirus B	2.4832
1511871	Viruses	Primate bocaparvovirus 1	0.0636
1335626	Viruses	Middle East respiratory syndrome-related coronavirus	1.379
147711	Viruses	Rhinovirus A	3.2147
11216	Viruses	Human respirovirus 3	8.2615
12730	Viruses	Human respirovirus 1	2.3088
1511872	Viruses	Primate bocaparvovirus 2	0.3509
162145	Viruses	Human metapneumovirus	0.1656

Table S2: Virus detection performance by adjusting the E-value cut-off on the simulated metagenomic dataset. Total number of viral contigs: 34. Total number of non-viral contigs: 173,955.

**Evalue cutoff**	**Type**	**pHMM Databases**						
		**EGG4.5**	**EGG5.0**	**vFam**	**pVOGs**	**Pfam**	**KOfam**	**VOGDB**
10	predicted	3977	9969	9062	9589	350	163	20083
	TP	34	34	30	6	20	1	34
	FP	3943	9935	9032	9583	330	162	20049
1	predicted	3829	9325	8334	9076	297	153	18994
	TP	34	34	30	5	20	1	34
	FP	3795	9291	8304	9071	277	152	18960
0.1	predicted	3714	8472	7608	8060	158	137	14222
	TP	34	34	30	4	20	1	34
	FP	3680	8438	7578	8056	138	136	14188
0.01	predicted	3548	6166	5726	5099	49	119	11181
	TP	34	34	28	4	20	1	34
	FP	3514	6132	5698	5095	29	118	11147
0.001	predicted	2992	5276	4742	4099	24	108	10039
	TP	34	34	27	3	20	1	34
	FP	2958	5242	4715	4096	4	107	10005
0.0001	predicted	2578	4752	4167	3593	21	91	9299
	TP	34	34	25	2	20	1	34
	FP	2544	4718	4142	3591	1	90	9265

Table S3: Virus detection performance by adjusting the model coverage cut-off on the simulated metagenomic dataset. Total number of viral contigs: 34. Total number of non-viral contigs: 173,955. Applying coverage cutoffs to filter shorter matches can effectively reduce the number of false positives. However, on all databases, this comes with the price of lower recalls, meaning that some viral sequences will be missed. Thus, this strategy help users who need to reduce FP hits but will not return the optimal tradeoff between recall and FP rate.

**Hit coverage cutoff**	**Type**	**pHMM Databases**						
		**EGG4.5**	**EGG5.0**	**vFam**	**pVOGs**	**Pfam**	**KOfam**	**VOGDB**
0	predicted	2992	5276	4742	4099	24	108	10039
	TP	34	34	27	3	20	1	34
	FP	2958	5242	4715	4096	4	107	10005
0.2	predicted	2499	4007	3889	3619	24	63	7875
	TP	34	25	26	3	20	1	32
	FP	2465	3982	3863	3616	4	62	7843
0.4	predicted	1673	2247	2187	2439	20	20	4420
	TP	28	21	21	3	20	1	27
	FP	1645	2226	2166	2436	0	19	4393
0.6	predicted	865	1231	1056	1488	18	4	2222
	TP	25	18	18	0	18	1	24
	FP	840	1213	1038	1488	0	3	2198
0.8	predicted	425	612	416	700	18	3	977
	TP	19	16	18	0	18	1	21
	FP	406	596	398	700	0	2	956
1	predicted	20	6	8	7	8	0	22
	TP	8	5	8	0	8	0	13
	FP	12	1	0	7	0	0	9

Table S4: Prediction results for different coverage cut-off combinations. Since each pHMM may cover distinct regions, we consider the union of the covered regions by different pHMMs to determine the total coverage of the query sequence. E-value cutoff (1.0E-04) is fixed. The notation [0.5, 0.8] signifies that a coverage threshold of > 0.5 on the contig sequence and > 0.8 coverage on the pHMM are both required. The coverage on a contig is calculated by considering the hits of multiple pHMMs on this target contig.

**Coverage combination**	**Type**	**pHMM Databases**						
		**Egg4.5**	**Egg5.0**	**vFam**	**pVOGs**	**Pfam**	**KOfam**	**VOGDB**
[0, 0]	predicted	1331	3261	2318	3477	755	256	6024
	TP	40	57	35	44	22	9	63
	FP	969	2383	1687	2583	542	179	4377
	Unknown	322	821	596	850	191	68	1584
[0.5, 0.5]	predicted	600	1211	795	1106	108	102	2447
	TP	32	49	24	31	15	5	56
	FP	426	869	562	808	68	70	1760
	Unknown	132	293	209	267	25	27	631
[0.6, 0.8]	predicted	380	836	540	694	82	66	1760
	TP	28	45	23	31	13	5	54
	FP	261	591	388	388	48	44	1262
	Unknown	91	200	129	129	21	17	444
[0.8, 0.8]	predicted	293	648	367	573	82	36	1265
	TP	23	43	19	29	13	3	51
	FP	200	448	256	410	48	25	901
	Unknown	70	158	92	134	21	8	303
[0.8, 1]	predicted	167	383	233	275	47	33	898
	TP	18	33	15	19	6	3	43
	FP	108	260	167	184	28	22	640
	Unknown	41	90	51	72	13	8	215
